# “HOW WILL I EVER KNOW I DIDN’T BRING IT ON MYSELF?”: PERCEIVED PERSONAL RESPONSIBILITY IN DEMENTIA RISK

**DOI:** 10.1093/geroni/igae098.1135

**Published:** 2024-12-31

**Authors:** Allie Peckham, Molly Maxfield, Dara James

**Affiliations:** Arizona State University, Phoenix, Arizona, United States; Arizona State University, Phoenix, Arizona, United States; Arizona State University, Tempe, Arizona, United States

## Abstract

As public health messages increasingly emphasize the importance of “lifestyle interventions” to reduce dementia risk, our study aimed to explore how community dwelling middle-aged and older adults perceive this information. Here, our focus is on understanding how information around lifestyle interventions influences individuals’ perceptions of their control over health in relation to dementia risk. Our study further investigated how these health-promoting interventions might unintentionally contribute to ‘lifestyle stigma’ for individuals diagnosed with dementia. Through a secondary framework analysis of 50 semi-structured interviews, we found that participants showed a sense of personal responsibility towards engaging in lifestyle interventions to lower their chances of dementia. To interpret our findings, we relied on the concepts of locus of control and attribution theory. Of the 50 participants, we discovered that 23 participants discussed dementia risk, and 13 felt a degree of personal responsibility for their dementia risk. Four believed they had personal responsibility and control and actively engaged in lifestyle interventions. The remaining nine participants also engaged in lifestyle interventions, aiming to find comfort in the potential event of a dementia diagnosis. Recognizing the multifaceted nature of dementia risk, including environmental and external factors beyond individual control, is essential to communicating effectively and combating ‘lifestyle stigma’ that is becoming increasingly associated with the condition. By acknowledging the interplay between individual, social, and societal factors, we can develop more inclusive and effective public health strategies and messages to reduce dementia risk and promote overall well-being.

